# Effects of high-intensity interval training on functional performance and maximal oxygen uptake in comparison with moderate intensity continuous training in cancer patients: a systematic review and meta-analysis

**DOI:** 10.1007/s00520-023-08103-9

**Published:** 2023-10-18

**Authors:** T. Neuendorf, R. Haase, S. Schroeder, M. Schumann, N. Nitzsche

**Affiliations:** 1 Department of Sports Medicine and Exercise Therapy, Chemnitz University of Technology, Chemnitz, Germany; 2 Department of Molecular and Cellular Sports Medicine, German Sport University Cologne, Cologne, Germany

**Keywords:** HIIT, MICT, Exercise, Therapy, Prehabilitation, Aftercare

## Abstract

**Introduction:**

High-intensity interval training (HIIT) is an appropriate training modality to improve endurance and therefore contributes to physical performance. This review investigates the effect of HIIT on functional performance in cancer patients. We reviewed the relative peak oxygen uptake (relV̇O_2PEAK_) and meta-analytical compared HIIT with moderate intensity continuous training (MICT). Furthermore, we took various training parameters under consideration.

**Methods:**

A systematic literature search was conducted in Scopus, PubMed, and Cochrane Library databases. For the review, we included randomized controlled trials containing HIIT with cancer patients. From this, we filtered interventions with additional MICT for the meta-analysis. Outcomes of interest were various functional performance assessments and V̇O_2MAX_.

**Results:**

The research yielded 584 records which fit the inclusion criteria, of which 31 studies with *n*=1555 patients (57.4±8.6 years) could be included in the overall review and 8 studies in the meta-analysis (*n*=268, 59.11±5.11 years) regarding relV̇O_2PEAK_. Different functional outcomes were found, of which walking distance (+8.63±6.91% meters in 6-min walk test) and mobility (+2.7cm in sit and reach test) improved significantly due to HIIT. In terms of relV̇O_2PEAK_, the performance of cancer patients was improved by HIIT (10.68±6.48%) and MICT (7.4±4.29%). HIIT can be favored to increase relV̇O_2PEAK_ (SMD 0.37; 95% CI 0.09–0.65; *I*^2^=0%; *p*=0.009). Effect sizes for relV̇O_2PEAK_ improvements correlate moderately with total training volume (Spearman’s ρ=0.49; *p*=0.03), whereas percentage increases do not (Spearman’s ρ=0.24; *p*=0.14).

**Conclusion:**

Functional and physical outcomes were positively altered by different HIIT protocols and forms of implementation, whereas a tendency toward more effectiveness of HIIT vs. MICT was found for relV̇O_2PEAK_. Future studies should include functional parameters more often, to finally allow a comparison between both training protocols in this regard.

**Supplementary Information:**

The online version contains supplementary material available at 10.1007/s00520-023-08103-9.

## Introduction

Adhering to common physical activity guidelines is considered an essential factor in prevention, treatment, and aftercare of various cancers [1], and has been shown to improve cancer-specific survival after treatments and all-cause mortality [2–4]. Exercise as a planned, structured, and repetitive subset of physical activity [5] has been shown to contribute to both the prevention and management for several chronic diseases [6], including cancer [7]. In addition to improved physical fitness and maintained activities of daily living, supervised physical training can make an impact on psychological well-being and consequently improve quality of life [7, 8]. Concomitant to medical treatment, exercise may be beneficial to reduce symptom experience (e.g., cancer-related fatigue) and other therapy-related symptoms (e.g., from radiation and pharmaceuticals), and the risk of recurrence can be reduced [7, 9–13].

Cardiovascular diseases (CVD) share a number of risk factors with cancer [14]. A study showed that CVD may be the primary cause of death in breast cancer survivors [15], an interesting finding that requires further evidence for other types of cancer. Studies show that cardiovascular training in cancer patients may be beneficial for multiple dimensions, such as physical function (e.g. V̇O_2_), cancer-related fatigue, and functional capacity [16–19]. This gives cardiovascular training (e.g., endurance exercise) a particular relevance for cancer survivors. Consistent with this, higher cardiorespiratory fitness has been associated with reduced cancer mortality [20]. However, while individualized endurance training is recommended as a part of an optimally designed exercise program in cancer patients [21], there is still a lack of consensus as to which type of endurance exercise is most effective. Endurance exercise can be performed continuously with low to moderate intensity (MICT) or intermittently [22]. High-intensity-interval training (HIIT) in particular, consisting of short, high-intensity training sessions (e.g., >80% maximal oxygen consumption [V̇O_2MAX_]) interspersed with low-intensity recovery phases [23, 24] has gained interest not only in elite sports but also in the therapy of various diseases [25–27]. Specific improvements were for example reduced dosage of medication and improved endurance performance in type 2 diabetes patients [25]. HIIT was found to be significantly more effective than MICT to improve cardiac functions in myocardia infarct patients [26].

Milanović et al. [28] found a potentially large positive effect on V̇O_2MAX_ of +5.5±1.2 ml kg^−1^ min^−1^ after HIIT compared to healthy controls who did not exercise in young to middle-aged healthy individuals. Moreover, HIIT may have additional benefits as it induces alterations in peripheral muscle tissue (e.g. increased fiber cross sectional area and capillary-to-fiber ratio) that lead to a reduction in adverse effects of training, such as dyspnea and leg discomfort [29]. In a comprehensive meta-analysis, Batacan et al. [30] report a significant improvement in V̇O_2MAX_ through HIIT in normal weight and overweight/obese populations, respectively. Furthermore, HIIT is a highly effective approach to improving cardiorespiratory fitness and quality of life in adults with chronic disease, especially in comparison with other forms of endurance training such as MICT [29, 31, 32].

As a result of early diagnosis and advanced treatment, cancer becomes a chronic disease for many people, with persistent side effects of therapy (e.g., loss of muscle mass and strength, loss of mobility and upper extremity disability, lymphedema, fatigue, and cardiac toxicity) [33]. Functional performance can be impaired by muscle loss, limited upper and lower extremity strength, reduced walking distance, and various physical symptoms [34–37] from which an essential goal in the cancer aftercare is derived.

Studies indicate that HIIT is more beneficial than MICT for improving functional performance and sustaining those effects after detraining [38, 39]. HIIT can therefore be an efficient training regimen to promote functional performance [38]. Superior effects of HIIT (vs. MICT) were also found for functional mobility in a healthy elderly population [39]. The application of HIIT is acknowledged to be feasible and safe for cancer patients and can be an alternative to conventional endurance training to increase physical capacity [40–42]. Due to the stated efficacy in terms of time, HIIT seems suitable for the supportive treatment of chronic diseases [43].

HIIT is a suitable form of training for a broad cancer patient population [42]. HIIT can be performed by various types of cancer in UICC stages I–IV in prehabilitation (e.g., [44]), therapy (e.g., [45]), and aftercare (e.g., [46]). Nevertheless, a combination of HIIT and chemoradiation therapy can lead to an exacerbation of side effects and the subsequent reduction in quality of life [47]. No substantial dropouts were reported even in a population with advanced cancer (stage IV) [48]. High adherence was documented regarding perceived training sessions and targeted intensities [46, 49].

While the positive effects of HIIT on physical fitness in cancer patients have been recognized [42], specific consideration of functional tests is lacking. Those outcomes could be essential to assess effects relevant to the everyday life of cancer survivors. In addition, HIIT protocols seem to be increasingly common in intervention studies from 2019 till now (total database records per year). Therefore, we performed a systematic literature review to analyze the functional performance following HIIT. We analyzed the effects of HIIT on maximal oxygen uptake (V̇O_2MAX_) and performed the meta-analytic approach comparing HIIT and MICT. In addition, we provide an overview of the specific features of the training programs used in the included studies. Based on the results of the review and meta-analysis, we aim to derive a possible preference regarding HIIT or MICT as a preferred training method in cancer patients.

## Methods

### Systematic literature search

**Fig. 1:**
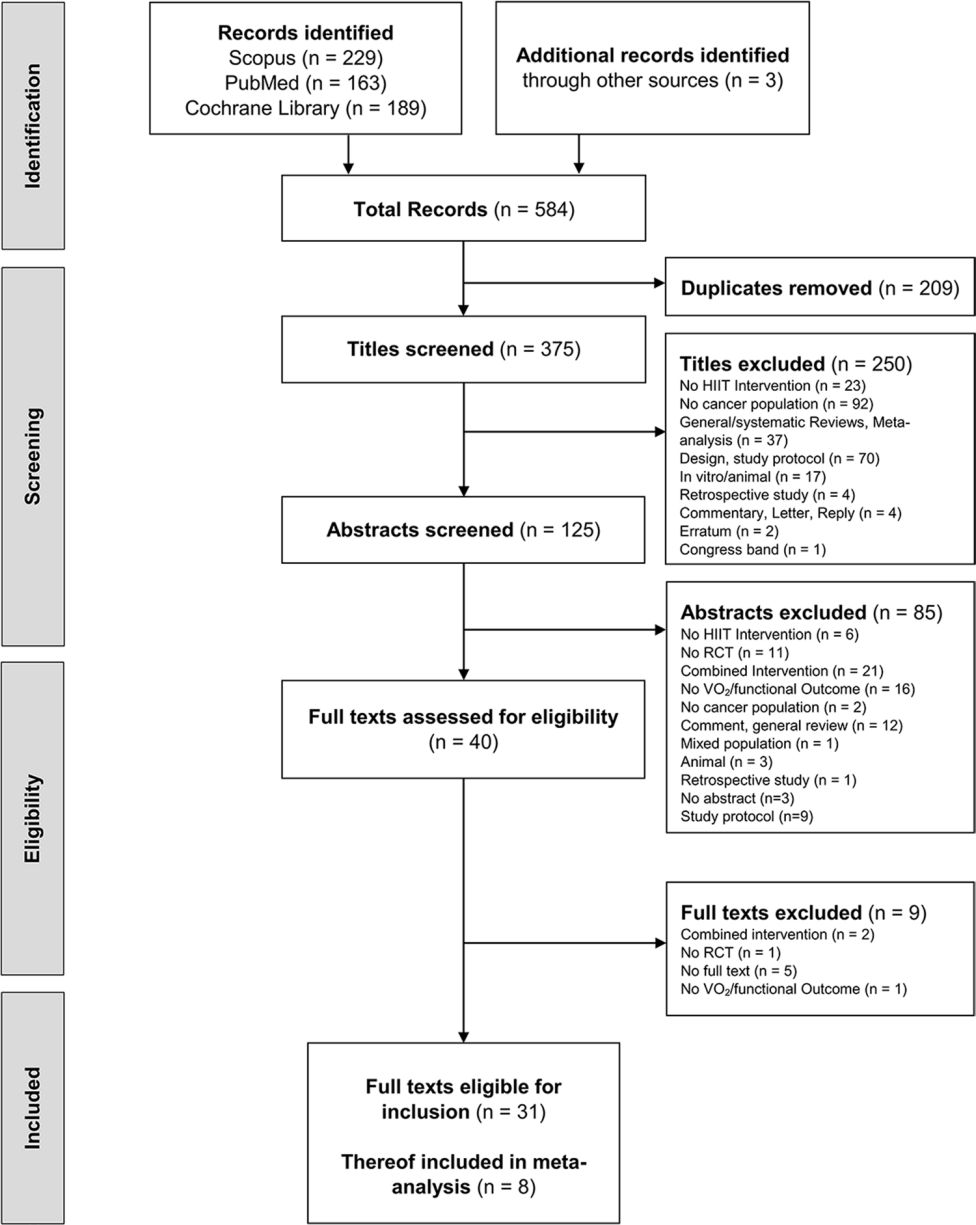
PRISMA flow chart to illustrate the selection of literature [70]

The research was performed in line with the PRISMA (Preferred Reporting Items for Systematic Reviews and Meta-Analyses) recommendations [50]. The search terms “cancer” AND “high intensity interval” were used for the systematic literature search. In March 2023, the PubMed, Scopus, and Cochrane Library databases were searched independently by two investigators using the specified search terms. In case of disagreement, a third reviewer was consulted. Information regarding the selection process is shown in the flow chart (Fig. 1).

### Eligibility criteria

The eligibility criteria were based on the PICOS framework (population, intervention, comparison, outcome, study design). Studies with adult cancer patients of all types of cancer, stage, and sexes were included. The intervention had to consist solely of HIIT (any interval intensities, durations, and frequencies) over a period of at least 3 weeks, with a control group receiving only medical treatment (e.g., no exercise training, usual care) or another group performing any form of MICT. Considered outcomes were various practical functional assessments (e.g., 6-min walk test (6MWT), timed up and go test (TUG), sit to stand test (STS), sit and reach test (SRT), grip strength (GS), Margaria-Kalamen stair test (MKST), and chair stand test (CST)) and measurements for cardiorespiratory fitness (CRF) V̇O_2PEAK_ or V̇O_2MAX_. We did not incorporate questionnaires, for instance on physical activity or self-assessments. Only randomized controlled trials (RCTs) with a pre-post design were included.

### Data extraction

The following data were extracted: (a) general study information—authors, publication year, study design; (b) subject information: sample size, anthropometrics, cancer-related information (e.g., usual care specifics, type of cancer , surgeries, time since diagnosis), UICC (Union for International Cancer Control) stages; (c) HIIT and MICT intervention data according to FITT criteria (F=Frequency; I=Intensity; T=Time; T=Type) [51]—duration, frequency, intensity, training equipment; (d) outcome parameter: functional assessments (e.g., 6MWT, sit to stand test, grip strength), measurements for CRF (relVO_2PEAK_, V̇O_2MAX_). Outcomes were extracted from pre- and post-data of the studies. This was followed by converting the outcome data into the respective percentage change of the parameter. In the case of unspecific data areas (e.g., 70–85%), the respective mean value was used for further calculations. We calculated a total training volume by multiplying training weeks by training frequency per week and the duration of one session in minutes.

If available, data was extracted in terms of mean, standard deviation (SD), and sample size for meta-analysis. If certain data was missing [52–54], we contacted the respective author for further details. Data of Devin et al. (2016) [54] was received and included into the analysis. If specific data was not presented numerically [52, 53], we extracted values from a figure by using the WebPlotDigitizer Tool [55]. Due to different ways of presenting and analyzing results, studies with seemingly identical samples were still included (Table 1) [46, 49, 56–58]. To categorize interval durations, we set three groups, i.e., ≤1min, 1–3min, and ≥3min, based on the diversity of the available data.

**Table 1:** Overview of included studies on the influence of HIIT training

Authors (year)	Sample	Cancer related supplementary information	UICC stage	Groups	Training duration in weeks	Training frequency per week	Training protocol	Training intensity	Training equipment	Outcome	Results
Adams et al., 2017 [46]	*n*=63 44 ± 11 years	Testicular cancer, cancer survivors: time since diagnosis on average 8 years	NI	Group 1: HIIT (*n*=35) Group 2: UC (*n*=28)	12	3	Group 1: 5min warm up, 4 × 4min incline walking or running, 3min rest, 5min cool down	Group 1: Warm up: 5% ventilatory threshold, intervals at 75–95% V̇O_2PEAK_ Active rest at 5–10% below ventilatory threshold	Treadmill	relV̇O_2PEAK_	Group 1: +4.2ml O_2_/kg/min Group 2: +0.6ml O_2_/kg/min HIIT vs. UC: *p*<0.001
Adams et al., 2018 [49]	*n*=63 44 ± 11 years	Testicular cancer, cancer survivors: time since diagnosis on average 8 years	NI	Group 1: HIIT (*n*=35) Group 2: UC (*n*=28)	12	3	Group 1: 5min warm up, 4 × 4min incline walking or running, 3min rest, 5min cool down	Group 1: Warm up: 5% ventilatory threshold, intervals at 75–95% V̇O_2PEAK_ Active rest at 5–10% below ventilatory threshold	Treadmill	relV̇O_2PEAK_	Group 1 vs. Group 2:+ 3.7ml O_2_/kg/min HIIT vs. UC: *p*<0.001
Alizadeh et al., 2019 [81]	*n*=50 Group 1: 49 ± 9 years Group 2: 48 ± 8 years	Breast cancer, completed therapy (chemotherapy, radiotherapy) in the last month prior to the intervention	I–III	Group 1: HIIT (*n* = 24) Group 2: CG (*n* = 26) (different information)	12	3	Group 1: Total 38min, 5min warm up, 4 × 4min walking uphill, rest 3min, 5min cool down Group 2: Normal physical activity levels	Group 1: HIIT:90–95% HR_MAX,_ Recovery_:_ 50–70% HR_MAX_	Treadmill	V̇O_2MAX_	Group 1: +21.65% HIIT vs. Control: *p*=0.002
Banerjee et al., 2018 [78]	*n*=60 Group 1: 72 ± 7 years Group 2: 73 ± 8 Years	Bladder cancer, preoperative, before radical cystectomy	NI	Group 1: HIIT (*n*=30) Group 2: CG (*n*=30)	3–6	2	Group 1: Warm up 5–10min, 6×5min, rest 2.5min	Group 1: Warm up: 50W, Borg 13–15 (ca. 70–85% predicted HR_MAX_, cool down at 50W	Cycling ergometer	relV̇O_2PEAK_	Group 1: 19.22±4.8 to 21.07±5.6 ml/kg/min Group 2: 20.38±5.59 to 20.84±5.43 ml/kg/min HIIT vs. Control: *p*=0.057
Bell et al., 2021 [89]*	n=20 Group 1: 49 ± 4 years Group 2: 51 ± 5 years	Breast cancer, completed therapy (chemotherapy, radiation therapy) at least 6 months prior	IA, IIA, IIB	Group 1: HIIT (*n*=10) Group 2: MICT (*n*=10)	12	2	Group 1: 3–5min warm up, 4×2min (weeks 1–2) 4×5min (weeks 3–12) Intervals, 5min cool down, 33min total Group 2: 5min warm up, 14 (weeks 1–2)–25min (weeks 3–12) continuous, 5min cool down, 33min total	Group 1: Warm up: Intervals at 70–75%HRR Group 2: 60%HRR	Cycling ergometer	relV̇O_2PEAK_	Group 1: 27.8±5.2 to 29.8±5.1 ml/kg/min Group 2: 26.3±7.0 to 27.4±7.0 ml/kg/min HIIT vs. MICT (*p*=0.21)
Bhatiaand Kayser, 2019 [56]	*n*=151 64 ± 12 years	Lung cancer, preoperative, before primary lung resection,	I–IIIA	Group 1: HIIT (*n*=74) Group 2: CG (*n*=77)	2-3	3	Group 1: 5min warm up, 2 × 20 × 15s, rest 15s, rest in between 4min, 5min cool down	Group 1: Warm up 50% W_PEAK_, intervals all out at 100% W_PEAK_ Pause: passive resting, cool down 30% W_PEAK_	Cycling ergometer	relV̇O_2PEAK_ 6MWT	Group 1: +14% (MED) CI 3–26 (*p*=0.004) of relV̇O_2PEAK_, +20% (MED) CI 14–26% of walking distance (*p*<0.001)
Blackwell et al., 2020 [44]	*n*=34 Group 1: 71 ± 2 years Group 2: 72 ± 4 years	Urological cancer, preoperative, before major urological surgery	NI	Group 1: HIIT (*n*=18) Group 2: UC (*n*=16)	4	3–4	Group 1: 5 × 60s Rest: unloaded cycling	Group 1: 100–115% (max load in W)	Cycling ergometer	relV̇O_2PEAK_	Group 1: MD +2.26 ml/kg/min (CI 0.24–4.08) (*p*<0.05)
Devin et al., 2016 [54]*	*n*=47 62 ± 11 years	Colorectal cancer, cancer survivors: time since diagnosis median=41 months	I–IV	Group 1: HIIT (*n*=30) Group 2: MICT (*n*=17)	4	3	Group 1: 10min warm up: 4×4min, rest 3min Group 2: 50min training	Group 1: warm up at 50–70% HR_MAX_, intervals at 85–95% HR_PEAK_ Pause: 50–70% HR_PEAK_ Group 2: 50–70% HR_PEAK_	Cycling ergometer	relV̇O_2PEAK_	Group 1: 24.7±8.1 to 28.2±7.5 mlO_2_/kg/min (*p*<0.001) Group 2: 23.3±5.4 to 24.2±6.4 mlO_2_/kg/min (*p*=0.245) HIIT vs. MICT: *p*=0.021
Devin et al., 2018 [74]*	*n*=57 61 ± 11 years	Colorectal cancer, stages I–IV, cancer survivors: time since diagnosis=4.1±2.5 years	NI	Group 1: HIIT (*n*=18) Group 2: HIIT (utilizing a tapered frequency prescription) (*n*=20) Group 3: MICT (*n*=19)	8	3 (Groups1 and 3) Group 2: 3 for weeks 1–4, followed by 1 for weeks 5–8	Groups 1 and 2: total 38min, 10min warm up, 4 × 4min, rest 3min Group 3: 50min	Groups 1 and 2: Warm up at 50–70% HR_MAX_, intervals at 85–95% HR_PEAK_, active rests Group 3: 50–70% HR_PEAK_	Cycling ergometer	relV̇O_2PEAK_	Group 1: +5.0 (mean) Group 2: +3.1 (mean) Group 3: +2.7 (mean) (all *p*<0.001) HIIT vs. MICT: +2.3 ml/kg/min (*p*=0.049) HIIT vs. group 2 (*p*>0.05)
Djurhuus et al., 2023 [65]	*n*=30 Group 1: 63 Group 2: 68	Prostate cancer, preoperative	NI	Group 1: HIIT (*n*=20) Group 2: CON (*n*=10)	2–8	4	Group 1: 10min warm up, 20–25min HIIT (4–6×1min, rest 3min Group 2: maintaining everyday lifestyle	Group 1: Warm up 30% W_PEAK_, 100–120% W_PEAK_, rest 30% W_PEAK_	Cycling ergometer	relV̇O_2PEAK_	Group 1: +0.8 (range −0.8–2.3) ml/kg/min (*p*>0.05) Group 2: +1.2 (range −1.1–3.5) ml/kg/min (*p*>0.05) HIIT vs. CON: −0.4 (range −3.6–1.9) (*p*>0.05)
Dolan et al., 2016 [73]	*n*=33 57,2 ± 9 years	Breast cancer, cancer survivors: time since diagnosis on average 6 years,	I–IIIA	Group 1: CG (*n*=10) Group 2: MICT (*n*=11) Group 3: HIIT (*n*=12)	6	3	Group 2: 3.22km (2mi)–4.02km (2.5mi) walking Group 3: Progressive interval training, 4–6×2–4min	Group 2: 55/60–70% V̇O_2PEAK_ Group 3: Intervals 65–95% V̇O_2PEAK_, rest 50–<60% V̇O_2PEAK_	Treadmill or outdoors (not specified)	relV̇O_2PEAK_	Note: Baseline based on total population Group 1: −-5.97±7.2% (*p*<0.001) Group 2: +12.95±10.4% (*p*<0.001) Group 3: +11,48±10,5% (*p*<0.001) MICT vs. Control (*p*<0.0001) HIIT vs. Control (*p*<0.0001)
Dunne et al., 2016 [79]	*n*=37 Median: 62 years	Liver cancer, preoperative, before liver resection	NI	Group 1: HIIT (*n*=20) Group 2: CG (*n*=17)	4	3	Group 1: Warm up, 30min HIIT, no details, cool down	Group 1: Intervals: ≥ 90% V̇O_2PEAK_ rest: ≤ 60% V̇O_2PEAK_	Cycling ergometer	relV̇O_2PEAK_	Group 1: 17.6±2.3 to 19.6±3.8ml O_2_/kg/min (*p*=0.019) Group 2: 18.6± 3.9 to 18.7±4.1ml O_2_/kg/min (*p*=0.958) HIIT vs. control (*p*=0.047)
Hooshmand Moghadam et al., 2021 [52]*	*n*=40 57 ± 1 years	Breast cancer, overweight and obese patients >6 months outside completion of cancer course treatment (mastectomy, lumpectomy, chemotherapy, radiation therapy)	I–III	Group 1: HIIT (*n*=13) Group 2: MICT (*n*=13) Group 3: CG (*n*=14)	12	3	20–30min total, 5min warm up, 5min cool down Group 1: 4–7×30s, rest 2min Group 2: 20min continuous Group 3: maintaining normal daily lifestyle	Group 1: Warm up at ≤50% PP, ≥90% HR_MAX_ after interval 4 Group 2: Warm up at ≤50% PP, followed by 55–65% PP	Cycling ergometer	relV̇O_2PEAK_	Group 1: +0.95 ml/kg/min (95% CI 0.68–1.21, *p*<0.001) Group 2: +0.67 ml/kg/min (95% CI 0.5–0.85, *p*<0.001) HIIT vs. MICT (*p*=0.178) No changes in CG
Hwang et al., 2012 [80]	*n*=24 Group 1: 61 ± 6 years Group 2: 59 ± 8 years	Lung cancer, during targeted cancer therapy (epidermal growth factor receptor inhibitors for >4 weeks)	IIIA, IIIB, IV	Group 1: HIIT (*n*=13) Group 2: CG (*n*=11)	8	3	Group 1: 10min warm up, 2–5min intervals (no details), 5min cool down (total 30–40min)	Group 1: Intervals at RPE 15–17 or 80% V̇O_2PEAK_, rest at RPE 11–13 or 60% V̇O_2PEAK_	Cycling ergometer or Treadmill	relV̇O_2PEAK_	Group 1: 15.1±3.4 to 16.8 ±4.1 ml O_2_/kg/min (*p*<0.005) Group 2: 16.7±4.8 to 16.3±4.6 mlO_2_/kg/min (*p*=0.27) HIIT vs. CG (time × group): *p*<0.005
Kang et al., 2021 [72]	*n*=52 63.4 ± 7.1	Prostate cancer, under active surveillance	T1c, T2a, T2b	Group 1: HIIT (*n*=26) Group 2: UC (*n*=26)	12	3	Group 1: 5min warm up, 5–8×2min, rest 2min, 5min cool down Group 2: no change in exercise level	Group 1: Warm up 60% VO_2PEAK_, Intervals 85–95% VO_2PEAK_, 40% VO_2PEAK_ rest, 30% cool down	Treadmill	relV̇O_2PEAK_ 6MWT TUG STS SRT	relV̇O_2PEAK_ HIIT: +0.9 (range 0–1.7) UC: −0.5 (range −1.4–0.4), HIIT vs. UC: 1.6 (CI: 0.3–2.9) (*p*=0.01) 6MWT: HIIT: +27m (CI 12–41) UC: +6m (CI −10–22) HIIT vs. UC: 20m (CI −2–41) (*p*=0.072) TUG HIIT: +−0s (CI −0.2 to 0.2) UC: −0.3s (CI −1.5 to 0.7) HIIT vs. UC: 0.6s (CI −0.4 to 1.7) (*p*=0.22) STS HIIT: +1 reps (CI 0 to 3) UC: +−0 reps (CI 0 to 2) HIIT vs. UC: + 1 reps (CI 0 to 3) (*p*=0.15) SRT HIIT: +2.7cm (CI −1.1 to 6.4) UC: −2.8cm (CI −5.6 to −0.1) HIIT vs. UC: 4.8 (CI 0.2 to 9.4) (*p*=0.042)
Karenovics et al., 2017 [57]	*n*=151 64 ± 12 years	Lung cancer, preoperative before lung resection	I–IIIA	Group 1: HIIT (*n*=74) Group 2: CG (*n*=77)	3.5	3	Group 1: 5min warm up, 2 × 20 × 15s, rest 15s, rest in between 4min, 5min cool down	Group 1: Warm up at 50% W_PEAK_, intervals all out at 100% W_PEAK_ Pause: passive resting, cool down 30% W_PEAK_	Cycling ergometer	relV̇O_2PEAK_	Group 1: +1.2ml O_2_/kg/min Group 2: −1.3ml O_2_/kg/min HIIT vs. CG: *p*=0.06
Lee et al., 2019 [45]	*n*=30 46.9 ± 9.8 years	Breast cancer, during anthracycline chemotherapy	I–III	Group 1: HIIT (*n*=15) Group 2: CG (*n*=15)	8	3	Group 1: 30min total, 7×60s Rest, 2min active recovery Group 2: current level of physical activity	Group 1: Intervals at: 90% PPO (highest power output generated during a maximal cycling) Pause: 10% PPO	Cycling ergometer	V̇O_2MAX_	Group 1: 19.7±8.7 to 19.4±6.6ml/kg/min (*p*=0.94) Group 2: 18.7±7.1 to 16.1±6.0ml/kg/min (*p*=0.001)
Lee et al., 2021 [83]	*n*=30 46.9 ± 9.8 years	Breast cancer, during anthracycline chemotherapy	NI	Group 1: HIIT (*n*=15) Group 2: CG (*n*=15)	8	3	Group 1: 30min total, 7×60s Rest, 2min active recovery Group 2: current level of physical activity	Group 1: Intervals at: 90% PPO (highest power output generated during a maximal cycling) Pause: 10% PPO	Cycling ergometer	6MWT TUG STS MKST	6MWT HIIT: +51m (*p*=0.05) CG: −6.59m (*p*=0.95) HIIT vs. CG: *p*=0.008 STS HIIT: +0.14 reps/30s (*p*=0.55) CG: −0.37 reps/30s (*p*=0.29) HIIT vs. CG: *p*=0.39 TUG HIIT: +0.06s (*p*=0.62) CG: +0.25s (*p*=0.28) HIIT vs. CG: *p*=0.52 MKST HIIT: 0.13s (*p*=0.12) CG: +0.32s (*p*=0.20) HIIT vs. CG: *p*=0.013
Licker et al., 2017 [58]	*n*=151 64 ± 12 years	Lung cancer, preoperative before lung resection	I–IIIA	Group 1: HIIT (*n*=74) Group 2: CG (*n*=77)	3.5	3	Group 1: 5min warm up, 2 × 20 × 15s, rest 15s, rest in between 4min, 5min cool down	Group 1: Warm up at 50% W_PEAK_, intervals all out at 100% W_PEAK_ Pause: passive resting, cool down 30% W_PEAK_	Cycling ergometer	relV̇O_2PEAK_ 6MWT	relV̇O_2PEAK_ Group 1: +2.9ml O_2_/kg/min, MED +14% (*p*=0.04) 6MWT Group 1: MED +15% (*p*=<0.001) relV̇O_2PEAK_ Group 2 −1.5ml O_2_/kg/min (MED −8%) (*p*=0.005) relV̇O_2PEAK_ HIIT vs. CG: *p*=0.004 6MWT HIIT vs. CG: *p*=0.001
Minnella et al., 2020 [53]*	*n*=42 64.5 ± 11.2 years	Colorectal cancer, preoperative	I-III	Group 1: HIIT (*n*=21) Group 2: MICT (*n*=21)	4	3	Group 1: 30min total, 5min warm up, 4×2min intervals alternated with 4×3min intervals, 5min cool down Group 2: 5min warm up, 30min continuous, 5min cool down	Group 1: 4×2min (85–90% peak power), 4×3min (80–85% PAT) Group 2: 80–85 PAT	Cycling ergometer	relV̇O_2PEAK_, 6MWT	Group 1: +1.95 (0.71–3.19) ml/kg/min (*p*=0.0049), +12.55m (range −7.83–32.92) Group 2: +0.45 (range −0.71–1.6) ml/kg/min (*p*=0.412), +18.07m (range −1.36–37.51) relV̇O_2PEAK_ HIIT vs. MICT (*p*=0.08) 6MWT HIIT vs. MICT (*p*=0.696)
Northey et al., 2019 [82]*	*n*=17 62.9 ± 7.8 years	Breast cancer, ≤24 months post diagnosis	I–III	Group 1: HIIT (*n*=6) Group 2: MOD (*n*=5) Group 3: CG (*n*=6)	12	3	Group 1: 20–30min (5min warm up, 5min cool down, 4–7×30s, rest 2min Group 2: 2min continuous Group 3: maintaining current lifestyle	Group 1: warm up at ≤50% PP, ≥90% HR_MAX_ after interval 4 Group 2: 55–65% PP	Cycling ergometer	relV̇O_2PEAK_	Group 1: VO2peak +10.3% (*p*<0.05) Group 2: VO2peak +5.6% (*p*>*p*.05) Group 3: VO2peak −2.6%, HIIT vs. MOD (*d*=0.19), HIIT vs. CG (*d*=1.28), MOD vs. CG (*d*=0.72)
Ochi et al., 2022 [84]	*n*=44 20–59 years	Breast cancer	I–IIA	Group 1: HIIT (*n*=21) Group 2: CG (*n*=23)	12	3	Group 1: 8×20s exercises, e.g., squat, lunges (no further information), 10s rest, 10min/workout (3min warm up, 4min training, 3min cool down), wearable to monitor physical activity Group 2: wearable to monitor physical activity, no further instructions	RPE 16–20	Home-based body weight exercises	relV̇O_2PEAK_ 6MWT GS CST	relV̇O_2PEAK_: HIIT: +0.9±1.7 ml/kg/min CG: −0.8±1.6 ml/kg/min HIIT vs. CG: 1.7 (range 0.7–2.7 (ES=1.06; *p*<0.01) 6MWT HIIT: +30±30m CG: 29±40m HIIT vs. CG: 1 (CI −21–23m) (ES=0.26; *p*=0.93) GS HIIT: +0.8kg CG: +0.4kg HIIT vs. CG: 0.4 (CI −0.9 to 1.6) (ES=0.26; *p*=0.53) STS HIIT: −0.9s CG: −0.4s HIIT vs. CG: −0.5 (CI −1.9 to 0.9) (ES=−0.21; *p*=0.50)
Piraux et al., 2021 [85]	*n*=72 69.1 ± 8.2 years	Prostate cancer, undergoing radiotherapy	KA	Group 1: HIIT (*n*=24) Group 2: resistance training (*n*=24) Group 3: UC (*n*=24)	5–8	3	Group 1: total 26–40min, 5min warm up 8–15×60s, 1min rest, 5min cool down Group 2: total 30min, 3×8–12 repetitions (targeting 8 major muscle groups with body weight, resistance bands, dumbbells)	Group 1: Warm up at 65–70% HR_MAX_, intervals at ≥85% HR_MAX_, rest at 65–70% HR_MAX_, cool down at 65–70% HR_MAX_	Cycling ergometer	6MWT	Compared to Group 3 (UC): Group 1: +7.5% (*p*=0.043) Group 2: +6.6% (*p*=0.042)
Piraux et al., 2022 [86]	*n*=18 MED: 62 (59.8–68.8) years	Rectal cancer, undergoing chemo radiotherapy	II–III	Group 1: HIIT (*n*=6) Group 2: resistance training (*n*=6) Group 3: UC (*n*=6)	5	3	Group 1: total 26–40min, 5min warm up 8–15×60s, 1min rest, 5min cool down Group 2: total 30min, 1–3×8–12 repetitions (targeting 8 major muscle groups with body weight, resistance bands, dumbbells)	Group 1: Warm up at 65–70% HR_MAX_, intervals at ≥85% HR_MAX_, rest at 65–70% HR_MAX_, cool down at 65–70% HR_MAX_	Cycling ergometer	6MWT	Group 1: +27.5m (+2.5%) Group 2: +24.5m (+4.2%) Group 3: +35m (+9.9%) Group 1 vs. group 2 vs. group 3 differences (*p*>0.05)
Reljic et al., 2022 [48]	*n*=27 55.4 ± 13.2 years	Different advanced cancers: colon/rectum (*n*=6), stomach (*n*=5), melanoma (*n*=3), liver (*n*=2), pancreas (*n*=1), esophagus (*n*=2), ovary (*n*=4), myelom (*n*=2), breast (*n*=2), lung (*n*=1) Ongoing anticancer therapy	III, predominantly IV	Group 1: HIIT (*n*=13) Group 2 (*n*=14): light physical mobilization exercises with electric stimulation below threshold that triggers a muscle contraction	12	2	Group 1: total 14min, 2min warm up, 5×1min intervals, rest 1min, 3min cool down Group 2: total 20min	Group 1: intervals at 80–95% HR_PEAK_, incrementally increasing Group 2: trunk flexion and extension, partial squats, butterfly movement, pull down movement	Cycling ergometer	relV̇O_2PEAK_	Group 1: +3.0ml/kg/min (*p*<0.001) Group 2: −0.9ml/kg/min
Samhan et al., 2021 [88]	*n*=60 Group 1: 49.7 ± 8.9 years Group 2: 48.9 ± 7.7 years	Breast cancer, overweight or obese patients, various ongoing therapy protocols: surgery (*n*=20), surgery+chemotherapy (*n*=25), surgery+radiotherapy (*n*=5), surgery+radiotherapy+chemotherapy (*n*=7), radiotherapy+chemotherapy (*n*=3)	I–III	Group 1: HIIT (*n*=30) Group 2: CON (*n*=30)	8	3	Group 1: 5min warm up, 4×4min intervals, 3min rest, 5min cool down Group 2: maintaining their daily routines, do not participate in any structured exercise program	Group 1: Warm up at 50–70% HR_MAX_, intervals at 75–90% HR_MAX_, rest at 50–60% HR_MAX_	Treadmill	relV̇O_2PEAK_	Group 1: +3.77ml/kg/min (*p*=0.002) Group 2: −0.5 ml/kg/min
Schmitt et al., 2016 [75]*	*n*=28 53.5 ± 9 years	Different cancer types, predominantly breast cancer, time since diagnosis 14±20 months	NI	Group 1: HIIT (*n*=14) Group 2: MICT (*n*=14)	3	3	Group 1: 5min Warm up, 8×60s fast walking, rest 2min slow walking Group 2: 60min walking outdoor + 15min indoor cycling 75min	Group 1: Warm up at 70% HR_MAX_, intervals at >95% HR_PEAK_ Group 2: 60% HR_PEAK_	Group 1: (walking outdoor, incline) Group 2 (outdoor, cycling ergometer)	relV̇O_2PEAK_	Group 1: 27.1±7.9 to 27.0±7.3 mlO_2_/kg/min (*p*=0.42) Group 2: 23.8±5.0 to 26.3±5.6 mlO_2_/kg/min (*p*<0.05) HIIT vs. MICT: *p*=0.01
Toohey et al., 2016 [76]*	*n*=16 52 ± 13 years	Various cancer types, cancer survivors <24 months after diagnosis after acute side effects due to cancer therapy	NI	Group 1: HIIT (*n*=8) Group 2: MICT (*n*=8)	12	3	Group 1: 5min warm up, 7×30s, rest 60s, 5min cool down Group 2: 5min warm up, 20min, 5min cool down	Group 1: Intervals ≥ 85% HR_MAX_ Group 2: ≤ 55% HR_MAX_	Cycling ergometer or treadmill	6MWT STS (time for 5 reps)	6MWT HIIT: 502.8±148.5 to 577.6±102.5m (18.53%) (*p*<0.05) MICT: 520.9±74.3 to 530.6±107.8m (1.16%) HIIT vs. MICT: partial eta^2^=0.5) STS HIIT: −6.39% MICT: −23.46% HIIT vs. MICT: partial eta^2^=0.06)
Toohey et al., 2018 [71]	*n*=75 51 ± 12 years	Various cancer types, cancer survivors <24 months after diagnosis	NI	Group 1: HIIT (*n*=25) Group 2: MICT(*n*=25) Group 3: CG (*n*=25)	12	3	Group 1: 5min warm up, 7×30s, rest 60s, 5min cool down Group 2: 5min warm up, 20min, 5min cool down	Group 1: ≥ 85% HR_MAX_ Group 2: ≤ 55% HR_MAX_	Cycling ergometer	6MWT STS (time for 5 reps)	6MWT HIIT: 510.7±114.9 to 607.7±85.5m (*p*<0.01, *d*=0.97) MICT: 483.1±72.3 to 518.6±94.5) (*d*=0.17) CG: 494.2±128.7 to 477.7±127.1 (*d*=−0.13) STS HIIT: 10.1±2.8 to 8.1±2.1 (*d*=−0.83) MICT: 10.6±2.8 to 9.2±2.0 (*d*=−0.59) CG: 9.6±2.3 to 10.49±2.7 (*d*=0.36)
Toohey et al., 2020 [77]	*n*=17 62 ± 8 years	Breast cancer, cancer survivors <24 months after cancer treatment	NI	Group 1: HIIT (*n*=6) Group 2: MICT (*n*=5) Group 3: CG (*n*=6)	12	3	Group 1: 7×30s, rest 2min active recovery Group 2: 5min warm up, 20min, 5min cool down	Group 1: W_MAX_ Group 2: 55–65% W_MAX_, adjusted during intervention, RPE 9–13	Cycling ergometer	relV̇O_2PEAK_	Group 1: 19.52 to 23.29 mlO_2_/kg/min (19.31%) (*p*=0.01) Group 2: 20.74 to 21.9 mlO_2_/kg/min (5.6%) Group 3: 20.9 to 20.36 mlO_2_/kg/min (−2.6%)
Wood et al., 2020 [87]	*n*=16 MED: 52 years (34–72)	Different types of leukemia, lymphona, myeloma Before allogeneic hematopoietic cell transplantation	NI	Group 1 (HIIT: *n*=6) Group 2: CG (*n*=10)	5–12 (week 3–12 included HIIT)	3–4	Group 1: 30min total, 5min warm up, 5×2min Intervals, rest 3min, wearables with goal to increase average steps/week Group 2: received no further instructions, also received wearables	Group 1: Intervals at ≥80% HR_MAX_	Home-based or local training resources: walking, jogging, running, cycling, elliptical, or stair climbing	relV̇O_2PEAK_ 6MWT	Results after MED 8.5 weeks relV̇O_2PEAK_ Group 1: +0.4ml/kg/min Group 2: +1.2ml/kg/min 6MWT: Group 1: +5.3m Group 2: −34.2m

*Studies included in meta-analysis; *HIIT* high-intensity interval training, *MICT* moderate intense continuous training, *CG* control group, *UC* usual care, *MED* median, *MD* mean difference, *CI* confidence interval, *ES* effect size, *relV̇O*_*2PEAK*_ relative peak oxygen uptake, *NI* no information, *PPO* peak power output, *PP* peak power, *HRR* heart rate reserve, *RPE* rate of perceived exertion, *6MWT* 6min walk test, *STS* sit to stand test, *TUG* timed up and go test, *MKST* Margaria-Kalamen stair test, *SRT* sit and reach test, *GS* grip strength, *CST* chair stand test

### Data synthesis and analysis

Only studies that analyzed a direct comparison of MICT and HIIT were included in the meta-analysis. Statistics, forest plot, and funnel plot were realized using RevMan (Review Manager Version 5.4, The Cochrane Collaboration, 2020) and IBM SPSS Statistics 29. Figure 4 was created using Grapher 12 (Golden Software). As all outcome measures were continuous variables, the intervention effects of each RCT were calculated using the standardized mean difference (SMD). A funnel plot was created to analyze symmetry and distribution for possible publication bias [59].

As the included RCTs differed in several aspects, the pooled effect size was calculated using the random-effects model, which is generally recommended [60] when heterogeneity between studies is assumed. The effect size of the change in V̇O_2PEAK_/V̇O_2MAX_ (SMD) was calculated using the following equation [61]:

$$\textrm{SMD}=\frac{{\overline{x}}_2-{\overline{x}}_1}{{\textrm{SD}}_{\textrm{POOL}}}$$

*x*_1_ and *x*_2_ are the sample means in the two groups [61]. The guideline values proposed by Cohen for the interpretation of the SMD are small (0.2), medium (0.5), and large (0.8) [62].

Heterogeneity between the included studies was assessed using the chi-square test and the *I*^2^ statistic. The *I*^2^ statistic determines the percentage of variability in the effect estimates that can be attributed to heterogeneity and can be interpreted as follows: 0–30% represents low heterogeneity, 30–60% represents moderate heterogeneity, and 60–100% represents high heterogeneity [63]. By pooling the SD values, a more accurate estimate of their joint value was obtained. SD_POOL_ was based on the SD from the baseline and the post values of the intervention group.

$${\textrm{SD}}_{\textrm{POOL}}=\sqrt{\frac{\left({n}_1-1\right)\times {S}_1^2+\left({n}_2-1\right)\times {S}_2^2}{n_1+{n}_2-2}}$$

*n*_1_ and *n*_2_ are sample sizes of each group, whereas *S*_1_ and *S*_2_ are the standard deviations in the two groups. In some cases (e.g., [52]), no SD was available for pre- and/or post-values. Standard deviations were consequently calculated using the standard error (SE) or confidence interval (CI):

$$\textrm{SD}=\textrm{SE}\times \sqrt{n}$$

Dividing the upper and lower limit of the CI by 3.92 was only used when a normal distribution could be assumed (e.g., large sample size) or was specifically reported. Otherwise, this value was based on a *t*-distribution (degrees of freedom −1, α = 0.05, two-tailed) [64].

$$SD=\sqrt{n}\times \left(\frac{\textrm{upper}\ \textrm{limit}-\textrm{lower}\ \textrm{limit}}{3.92}\right)$$

The SE of the SMD is the square root of the variance (*V*_D_) of the SMD [61]:

$${V}_D=\frac{n_1+{n}_2}{n_1\times {n}_2}+\frac{SMD^2}{2\times \left({n}_1+{n}_2\right)}$$

Furthermore, we conducted a correlation analysis between the change in V̇O_2PEAK_/V̇O_2MAX_ (% change, effect size (ES)) and the total exercise volume within the intervention group (HIIT).

Due to a violation of the assumption of normal distribution, a rank correlation (Spearman’s rho (ρ)) was used and interpreted according to Cohen [62]. As a positive correlation between the number of training sessions and V̇O_2PEAK_ was already shown [65], we hypothesized an improvement in V̇O_2PEAK_/V̇O_2MAX_ and therefore performed a one-tailed correlation test. Statistical significance was assumed at *p*<0.05.

### Study quality and risk of bias assessment

Study quality was assessed using the Tool for the assEssment of Study qualiTy and reporting in EXercise (TESTEX). TESTEX is a 15-point scale and includes 5 points for study quality and 10 points for study reporting. This assessment tool was specifically designed for use in exercise training studies. A high total score indicates high study quality [66]. FITT and TESTEX have already been used in the context of cancer and exercise (e.g., [67]).

**Fig. 2:**
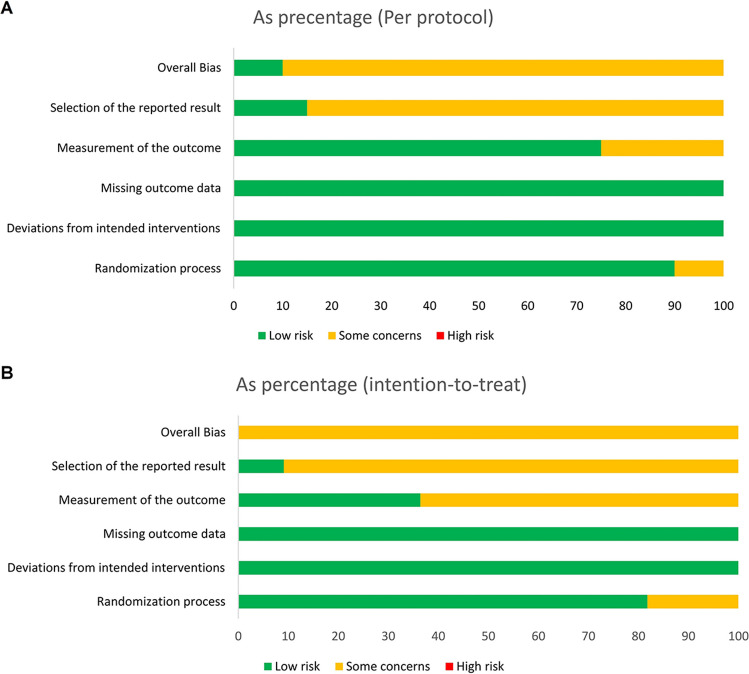
Risk of bias analysis: **A** per protocol (*n*=20), **B** intention to treat (*n*=11)

In addition, the revised Cochrane risk-of-bias tool [68] for randomized trials was used interdependently by two assessors to assess risk of bias. Five domains each relate to different aspects of bias: 1, randomization process; 2, deviations from the intended interventions; 3, missing outcome data; 4, outcome measurement; 5, selection of the reported outcome. To support the assessment of bias risk for the domain, the respective signal questions were answered and algorithms were followed to link the answers to the signal questions with suggestions for the resulting bias risk assessment [69]. The evaluation results of the publications consulted and evaluated for this work are shown (Fig. 2).

## Results

The literature search resulted in a total of 584 records. A total of 209 titles were not included due to duplication. The remaining 372 titles were screened with regard to title and abstract. After further exclusions, 31 publications were finally included in the review. Due to the lack of adequate study data, no meta-analysis could be performed regarding the functional outcome. Eight studies were included in the meta-analysis of relV̇O_2PEAK_ (Fig. 1).

The studies achieved an average total score of 11.6±1.3 (9–14) on the TESTEX scale. The average study quality was assessed high, with 4.0±0.9 (2–5) points, the study reporting dimension was moderately high, with 7.6±1.2 (5–9) points (see Supplementary Table). Therefore, no study had to be excluded due to poor study quality.

Based on the risk of bias analysis, the included studies were not found to be high risk. Only domain 5 “Selection of the reported result” was ranked having “some concerns,” as the relevant information could not be obtained from the available publications (Fig. 2).

### Study population

A total of *n*=1555 patients aged 57.4±8.6 years were included in the review. A total of *n*=268 patients aged 59.11±5.11 years were included in the meta-analysis. Cohorts with different cancer types (e.g., [71]) or a specific indication (e.g., prostate cancer [72]) were studied. The population varied between 16 and 151 cancer patients. The patients were at different diagnostic stages of cancer (UICC stages I–IV). When participating in the respective interventions, 8 years [46, 49], 6 years [73], 4.1 years [74], 1–2 years [75], and ≤ years [71, 76, 77] elapsed after cancer diagnosis, respectively. In some cases, training was used preoperatively (e.g., cystectomy, lung resection, liver resection) [44, 56–58, 78, 79]. In two studies, training was performed in-treatment, concomitantly to usual care, such as chemotherapy [45] or epidermal growth factor receptor inhibitor therapy [80]. In one study, patients participated in the HIIT intervention within the aftercare period shortly after completing chemotherapy and radiotherapy [81].

### Study design

Twenty-three of the selected publications comprised a two-armed design. Eight studies included a three-armed design [52–54, 71, 73–77, 82]. Patients were assigned to an intervention group (HIIT) or a control group (e.g., UC, MICT). Twenty-four studies compared HIIT with UC [44–46, 49, 52, 56–58, 65, 71–73, 77–88], 9 compared HIIT with MICT [52–54, 71, 73–77, 82, 89].

### Functional performance

Functional outcomes were assessed in 12 studies [53, 56, 58, 71, 72, 76, 77, 83–87]. All of these included the 6MWT, either as the only functional assessment or in combination with others. Significant improvement after HIIT was shown in seven cases [56, 58, 71, 72, 76, 83, 85], six of which documented improved walking distance and one of which had a significant positive change in mobility [72]. Functional performance based on walking distance (6MWT) increased by 8.63±6.91% (range 1.73 to 19.02%) after HIIT and by 4.61±3.88% after the continuous method. Two studies analyzed the increase in maximal walking distance and additionally compared HIIT and MICT: The results show a distinct superiority of the respective HIIT group (+18.53% HIIT vs. +1.16% MICT [76]; +19.02% HIIT vs. +7.35% MICT [71]). No further meta-analytic approaches could be derived.

### Physical performance and meta-analysis

RelV̇O_2PEAK_ and V̇O_2MAX_ were assessed as the outcome in 24 [44, 46, 48, 49, 52–54, 56–58, 65, 72–75, 77–80, 82, 84, 87–89] and 2 [45, 81] studies, respectively (Table 1). HIIT increased relV̇O_2PEAK_ by 10.68±6.48%, while MICT led to improvements of 7.40±4.29% (range −0.37 to 22.41%). A significant improvement after HIIT was shown either in an improvement in the relV̇O_2PEAK_ or V̇O_2MAX_ from pretest to posttest [44, 46, 49, 54, 56–58, 73, 74, 77, 79–81]. Several times the respective between group differences showed a statistically significant improvement after HIIT, compared to UC [46, 49, 57, 58, 72, 79–81, 83–85] (Table 1).

**Fig. 3:**
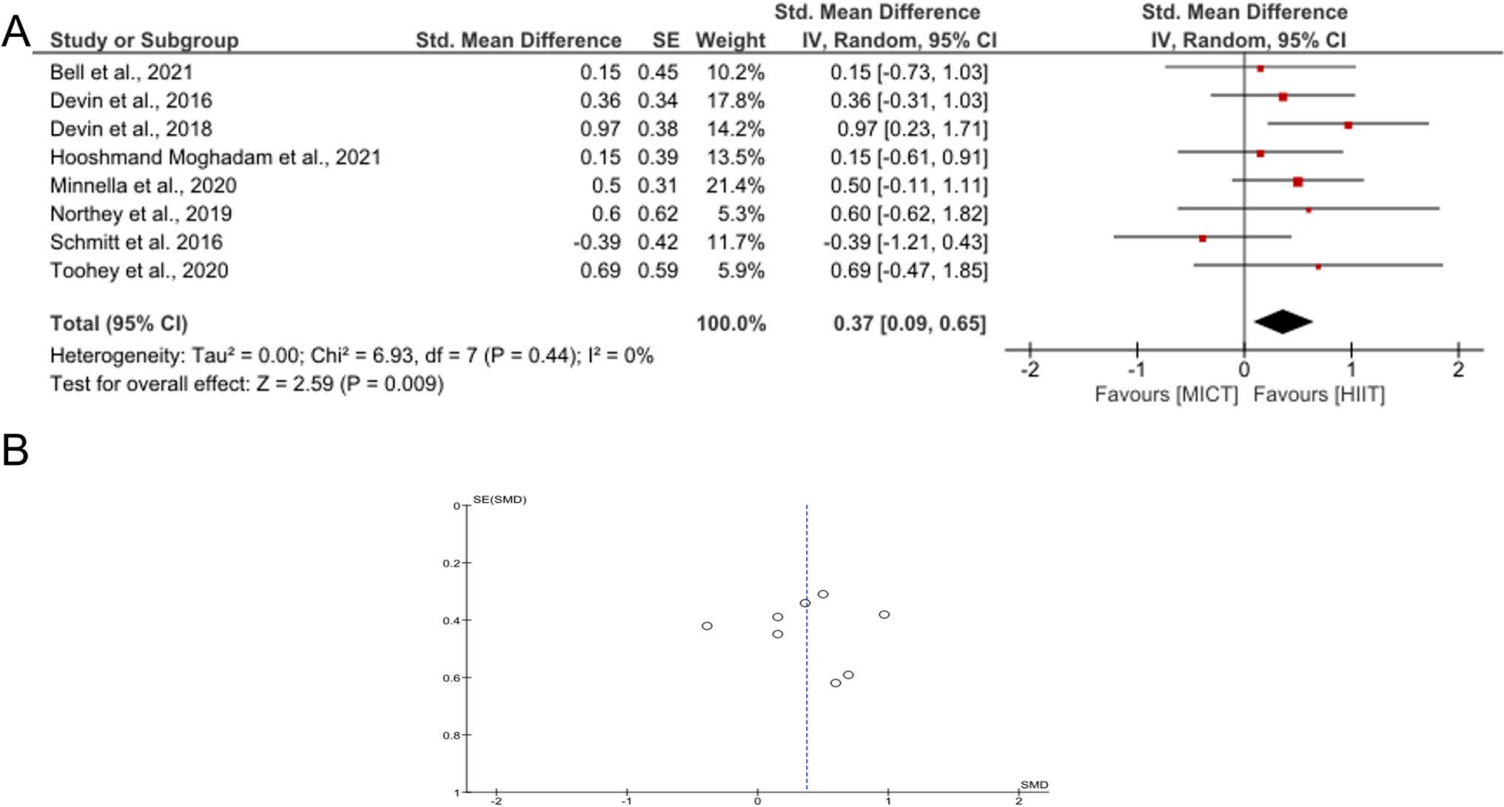
Forest plot to compare HIIT and MICT regarding relV̇O_2PEAK_ (**A**) and funnel plot to evaluate publication bias (**B**)

Eight studies were analyzed for the effects of MICT versus HIIT on the increase in oxygen uptake [52–54, 74, 75, 77, 82, 89]. The meta-analysis showed that HIIT had a small but significant main effect (SMD=0.37; CI 0.09–0.65; *p*=0.009). One study showed a preference toward the MICT method [75], while another [73] did not favor either form of training. The heterogeneity between the studies was *I*^2^ = 0% (Fig. 3A). Due to its symmetry and data distribution, the funnel plot indicates no strong publication bias (Fig. 3B). Dolan et al. [73] had to be excluded from the meta-analysis because of missing baseline data (values were only presented for the entire population). Therefore, no specific effects for either HIIT or MICT could be calculated.

### Training protocol and parameters

The duration of the training intervention was 8.0±3.6 weeks and varied between 2.5 and 12 weeks. Patients completed a mean of 3.0±0.4 training sessions per week (Table 1).

**Fig. 4:**
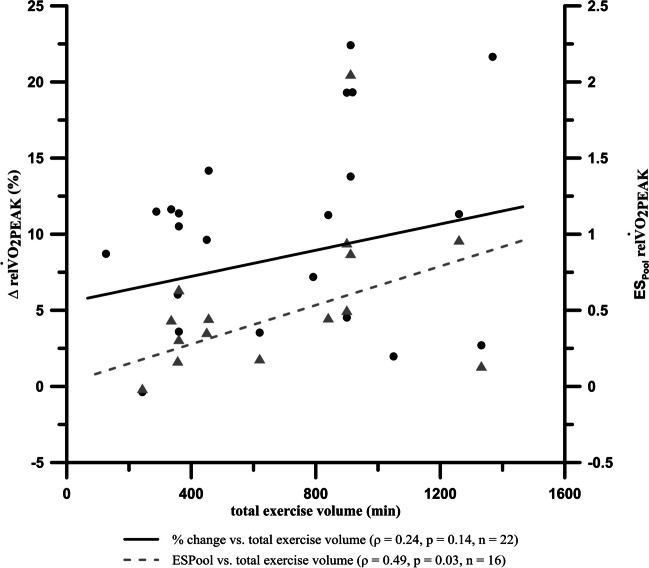
Rank correlation (Spearman’s rho (ρ)) between relV̇O_2PEAK_ and total exercise volume (dots and solid line) as well as rank correlation between ES_POOL_ of pre to post values of relV̇O_2PEAK_ and total exercise volume (triangles and dashed line); total exercise volume = training weeks × training frequency per week × duration of one session; note: only 15 triangles are visible due to overlay

The 31 publications differed regarding the duration of intervals and rest, or different ratios of loading and unloading durations. The analyzed studies used interval durations between 0.25 and 5 minutes. The average interval length was 1.9±1.6 min. Seventeen studies implemented an interval of ≤1min [44, 45, 48, 52, 56–58, 65, 71, 75–77, 82–86], 10 studies used intervals of ≥3min duration per interval [46, 49, 54, 73, 74, 78, 80, 81, 88, 89], and three studies used intervals between 1 and 3min [53, 72, 87]. The duration of MICT (excluding warm up and cool down) was 31.50±15.64min (20–60min). Only one study reported a distance in miles [73]. The intervals were repeated 9.5±10.6 times (range 4–40 times). Two studies reported no information on specific interval design [79, 80]. The correlation between effect sizes regarding relV̇O_2PEAK_ showed a significant moderate relationship (Spearman’s ρ=0.49; *p*=0.03) with total training volume, whereas percentage increase showed no significant correlation (Spearman’s ρ=0.24; *p*=0.14). If Kang et al. [74] is excluded from the correlation analysis as an inconsistent outlier due to an exceptional low effect while applying a high total training volume, we can state a moderate to high correlation (Spearman’s ρ=0.75; *p*<0.01) for the remaining 15 studies. Due to missing data (e.g., missing SD), it was not possible to calculate SD_POOL_ for every study. Finally, we calculated 22 data points for percentage change relV̇O_2PEAK_ [44, 46, 48, 52–54, 57, 65, 72–75, 77–82, 84, 87–89] and 16 data points for ES_POOL_ [46, 48, 52–54, 57, 65, 72, 74, 75, 78–80, 82, 84, 88] (Fig. 4).

In addition, different training intensities were applied. Nine of the 31 included publications selected power-related intensity parameters, for example 100–120% of the maximum power achieved during the pre-exercise cardiopulmonary exercise test (CPET baseline) or 90% peak power output [44, 45, 53, 56–58, 65, 77, 83]. Five of the 31 included studies used intensities based on V̇O_2_ values achieved during CPET and trained at 75–95% relV̇O_2PEAK_ [46, 49, 72, 73, 79]. Fourteen studies used 85–95% of maximum heart rate (HR_MAX_) as the exercise intensity in the HIIT intervention groups [48, 52, 54, 71, 74–76, 81, 82, 85–89]. Further studies used subjectively perceived exertion [78, 84], VO_2PEAK_, or subjectively perceived exertion [80].

HIIT was performed on the cycle ergometer (*n*=20) or treadmill (*n*=5), a combination of cycling ergometers or treadmills (*n*=2), using body weight exercises (*n*=1), training outdoors (*n*=1), or a free selection of multiple devices (*n*=1) (Table 1). One study did not provide any specific information [73].

Schmitt et al. [75] organized the training outside on an uphill road with short walking breaks between intervals. The interval training in Dolan et al. [73] started with low-intensity intervals (65–75% relV̇O_2PEAK_) for the first 2 weeks and increased progressively to 80–95% relV̇O_2PEAK_. As the intensity increased, the duration of the intervals decreased from 4 to 2 min.

## Discussion

The aim of this systematic review was to determine the effects of HIIT on the functional outcome and relV̇O_2PEAK_ in cancer patients. In addition, a meta-analytic approach compares HIIT vs. MICT regarding relV̇O_2PEAK_. We also provide a detailed overview of implemented training parameters.

### Functional outcomes

The functional outcomes walking distance (6MWT), mobility (SRT), grip strength (GS), and lower extremity strength (STS) were found in the reviewed studies. The data at hand suggest that an intervention with HIIT can significantly improve walking distance [56, 58, 71, 76, 83, 85] and mobility [72] in cancer patients. When HIIT was performed with strength-oriented body weight exercises, grip strength improved twice as much compared to UC [84]. This indicates that the method and manner HIIT is implemented may determine possible functional outcomes. It is plausible that GS would not be altered after HIIT, riding a stationary bike where no specific GS is required. In some cases, functional parameters such as TUG and STS remained almost unchanged following HIIT so that no differences from the control group can be observed [72, 83]. Toohey et al. [71, 76] depicted opposing results for STS in a comparison between HIIT and MICT: In one study, the MICT group showed stronger improvements [76] whereas the another study [71] presents a stronger effect after HIIT.

The resulting walking distance can be used as a marker of aerobic fitness and as a protective factor for cancer mortality [90] and therefore is of great relevance. Accordingly, it is particularly significant for cancer patients to achieve practically relevant improvements in walking distance as a benefit of HIIT. Walking distance is also related to health status in cancer patients (quality of life, cancer-related symptoms) [91]. In addition, the 6MWT could be used to plan and control training intensities, for example in the context of HIIT [92]. The observed improvements [56, 58, 71, 72, 76, 83–86] exceeded the minimal clinically meaningful difference for multiple patient groups (including cancer survivors) of 14–30.5m [93] or 62.5m [94] improvements, respectively. Nevertheless, contrary results were also found for walking distance, HIIT resulted in marginal gains [87], and MICT resulted in greater improvement [53].

Due to lack of a specific comparison between HIIT and MICT, no meta-analysis for functional outcomes could be performed. Toohey et al. [71, 76] presented 6MWT data for both training protocols that suggested a superiority of HIIT. We found more 6MWT [53, 56, 58, 72, 83–87] and other functional outcomes [71, 72, 76, 83, 84], but none of these studies compared HIIT vs. MICT. Although specific evidences for cancer patients are limited, our meta-analytic data are in line with found differences between HIIT and MICT in healthy elder populations: Coswig et al. [38] found greater improvements in STS and 6MWT after HIIT (vs. MICT) in elderly women. Coetsee and Terblanche [39] presented greater functional improvements in TUG after HIIT (vs. MICT) in a healthy older population. Our results suggest that HIIT has the same tendency to improve functional performance, but further studies need to address the direct comparison between HIIT and MICT to verify these findings.

### Physical outcomes

The review of all studies showed a rather clear superiority of complementary HIIT compared to UC [46, 49, 57, 58, 72, 79–81, 83–85]. Control groups receiving usual care alone showed a partial decrease in relV̇O_2PEAK_ over the intervention period [46, 52, 57, 58, 72, 73, 78–80, 82, 84, 88]. We found that both HIIT and MICT were shown to improve physical performance in patients across all cancer stages I–IV. Effects with HIIT occurred despite different training protocols (intervention duration, training frequency, training volume, or training intensity).

As shown in the meta-analysis, 7 out of 8 studies are presenting pronounced results after HIIT in terms of relV̇O_2PEAK_ (SMD 0.37; 95% CI 0.09 to 0.65; *I*^2^=0%; *p*=0.01); we can conclude superiority of the HIIT modality vs. MICT (Fig. 3). Mugele et al. [42] found no clear superiority of HIIT compared to MICT for relV̇O_2PEAK_ (MD 1.36; 95% CI −1.62 to 4.35; *p*=0.37). Due to a greater data source to evaluate this comparison, we conclude that HIIT may be more beneficial that MICT in order to improve relV̇O_2PEAK_. Hooshmand-Moghadam et al. [52] also concluded that HIIT is more beneficial than MICT for improving physical fitness (here: relV̇O_2PEAK_ + low body strength).

Different methods to average peak values (e.g., over 20s or 30s) were used to determine relV̇O_2PEAK_/V̇O_2MAX_, which limits the direct comparability of the data [95–99]. The extent to which V̇O_2MAX_ can be achieved with in patients is debated [100, 101]. In many cases, a symptom-limited V̇O_2PEAK_ is assumed to be lower than the actual V̇O_2MAX_ [100]. Most studies included in this review reported the relV̇O2peak. Of the reviews studies, only Alizadeh et al. [81] and Lee et al. [45] acclaimed having achieved a V̇O_2MAX_. Alizadeh et al. [81] estimated the V̇O_2MAX_ using a submaximal test (Rockport 1 mile walk test) while Lee et al. [45] determined V̇O_2MAX_ using a ramp test on a cycling ergometer but did not provide information on criteria for workload. In general, when leveling off is reached, it is assumed that exhaustion and V̇O_2MAX_ are reached [98, 100, 101]. Thus, it should be taken into account that, if necessary, patients did not reach a leveling off and relV̇O_2PEAK_ values were collected here.

When interpreting the aforementioned results, it should be noted that they only indirectly reflect the effects for the individual. It is therefore possible that the HIIT training protocol can achieve significantly higher, but also lower functional or physical effects in individual cases. Partially contradictory results are shown, for example, in the study by Boereboom et al. [102], in which individuals show strong positive changes in oxygen uptake, while others show negative changes. A clear attribution of cause (e.g., dependence on baseline level, number of training sessions performed) was not given. It is plausible that novice or, as in this case, deconditioned patients show significantly higher individual training effects than a person experienced in training [103]. In some cases, novices without experience with intensive training [46, 49] or inactive patients (did not achieve guideline recommendations for moderate or intensive activity) were explicitly included [71, 78].

### Training parameters and implementation

We stated that HIIT was performed on both the treadmill [46, 49, 72, 76, 80, 81, 88] or the cycling ergometer [44, 45, 48, 52–54, 56–58, 65, 71, 74, 76–80, 82, 83, 85, 86, 89]. In two cases, training was performed by walking outdoors [73, 75]. Since running promotes greater muscle mass than cycling, the working muscle mass used differs between the “running” and “cycling” forms of exercise, limiting a direct comparison [104]. HIIT on the treadmill was shown to result in higher heart rate and oxygen uptake than the same exercise on the cycling ergometer in healthy individuals [105]. It is possible that the high-intensity loads outside (weather, ground conditions, elevation profile) compared to controlled laboratory conditions (treadmill, cycling ergometer) may have an impact on the target parameters. Two studies differed by applying home-based HIIT: Exercises were performed outside or at local training resources in various forms of endurance training [87] or using the patients’ own body weight [84].

A respective HIIT design was approached and implemented differently by the authors of each study. Thus, interval durations varied from 0.25 to 5 min (mean 2.2±1.8 min) and were repeated 4–40 times (mean 12±13.7). Furthermore, it is important to question the extent to which the load design of 4×4min [46, 49, 81] or 6×5min [78] still fulfills the characteristics of short, high-intensity intervals of HIIT. In this review, we observed a differing values to measure training intensity (%HF_MAX_, % relV̇O_2PEAK_) (Table 1). Other studies used other data (e.g., % peak power output). Intensities ≤80% were documented that, according to the definition by MacInnis et al. [106] (≥80% HR_MAX_, mostly 85–95% HR_MAX_), they did not correspond to the definitions of HIIT (e.g., 90). In some cases, ranges of ≤80 to ≥80% were also reported [46, 49, 73, 78, 88] (Table 1).

Schlüter et al. [107] compared 10×1min vs. 4×4min HIIT acutely protocols at 85–95% HR_MAX_ (breast and prostate cancer patients) and concluded that a 4×4min protocol induced a higher energy expenditure and higher cardio-circulatory and metabolic strain. Therefore, if a high training stimulus is intended, a longer interval duration is preferable. However, an instructing physical therapist has to supervise if the patients tolerate rather long intervals, especially when undergoing therapy. Low training experience could also be a limiting factor in order to maintain intense intervals for several minutes.

A meta-analysis by Bacon et al. [108] showed that the design of the load factors during HIIT has an influence on the results in healthy individuals. It seems that especially the duration of the intervention in weeks is decisive. To take that into account, we included this parameter in total training volume.

A major criteria and possible promise of HIIT (compared with MICT) is generating relevant effects in a short time through short, intense intervals. Therefore, we analyzed the correlations between effects of HIIT on relV̇O_2PEAK_ and total training volume (Fig. 4). We are aware of possible confounding factors that have been considered with regard to the reliability of this statement, yet we selected a specific training parameter directly in the context of HIIT. The analysis indicates no direct dependence of total training volume and effects on relV̇O_2PEAK_ (Fig. 4). HIIT appears to be suitable for cancer patients to achieve relevant effects on endurance performance even in a short but intensive training period, although Lavín-Pérez et al. [109] point out that the exercise level should be at least 8 weeks, 2×/week (of which 15min HIIT/week) in cancer patients to achieve the highest return in health-related quality of life. In addition to intensity, it is possible that the total training volume represents a decisive parameter for training management of HIIT in cancer patients.

Yet, every implementation of HIIT has to be depending on the individual physical capabilities which may be altered due to timing during therapy or aftercare respectively. Even though the data indicate that HIIT is beneficial and helpful in improving performance, it still represents an intensive form of endurance exercise, where the patient’s health condition has a limiting effect on its applicability. A patient undergoing treatment may suffer from side effects, while the performance in aftercare may be impaired due to long-term cancer therapy and management. An individualized and supervised training regimen, in which specific training parameters can be modulated, could be key to implementing HIIT in prehabilitation, during treatment and aftercare as well. There is no specific red flag that excludes HIIT in any stage. A regression analysis of the influence of training volume and intensity could not be performed due to the partially imprecise or missing indications of the achieved training intensity (ranges from-to) (Table 1). As mentioned earlier, the study by Dolan et al. [73] was included even though the authors chose a progressive increase in intensity. This should be taken into account when interpreting the results and could be one reason why interval training was not superior to the continuous method in terms of relV̇O_2PEAK_ improvement, in contrast to other studies.

Based on this experimental application of the HIIT training form, insights for further therapeutic practice can be derived and aspects of the suitability and practical implementation of HIIT can be specified. In the context of endurance training in cancer patients, interval training can be used as a suitable, tolerable form of exercise. This is especially true, if a continuous load without breaks and over a longer period of time is not yet tolerated. HIIT is a suitable form of endurance training to improve cancer-related fatigue [49, 110]. Taking into account the shorter “economic” training time, HIIT may be sufficient to contribute to the prevention of cardiovascular events or the reduction of cancer related fatigue. HIIT may thus represent an important contribution to improving physical fitness and health-related outcomes, and may add significant value compared to usual care [42].

## Summary and outlook

The review showed that different functional outcomes were positively altered through HIIT. Our data indicates that HIIT might be more effective than MICT. Because functional outcomes were often not considered in the reviewed HIIT studies, no meta-analytic approach could be realized regarding the functional outcomes. We suggest that more attention should be paid to the functional outcome component to enable further direct comparisons between HIIT and MICT in terms of outcomes that are highly relevant to the daily lives of cancer survivors.

Furthermore, this review showed that positive changes in relV̇O_2PEAK_ were achieved with both MICT and HIIT, with HIIT usually having greater effects. Usual care alone mostly led to a decrease in performance. Results of the meta-analysis showed that HIIT appears to have greater effects on relV̇O_2PEAK_ compared to MICT. Further studies are needed to verify these results for relV̇O_2PEAK_.

Precise information on frequency, duration, and intensity of the respective intervals cannot yet be given but could be optimized by the respective trainer in the future. Distinct relationships with various exercise factors (e.g., duration, intensity, frequency) have to be addressed in a targeted and systematic manner. Furthermore, the application of HIIT in the real clinical setting of cancer therapy should be verified. The present “black box” about how HIIT is implemented should be analyzed with concrete application-related data from clinical practice.

## Supplemementary information

ESM 1
