# Supplementary material for: Effects of high-intensity interval training on functional performance and maximal oxygen uptake in comparison with moderate intensity continuous training in cancer patients: a systematic review and meta-analysis
Source: Support Care Cancer. 2023 Oct 18;31(11):643. doi: 10.1007/s00520-023-08103-9 (PMC10584719; doi:10.1007/s00520-023-08103-9)
Supplement: Supplementary file 1 — ESM 1 [file 520_2023_8103_MOESM1_ESM.docx]

*Tab. 1: Results of the TESTEX Analysis*

| **Study** | **Study quality criteria** | | | | | | **Study reporting criteria** | | | | | | | | |
| --- | --- | --- | --- | --- | --- | --- | --- | --- | --- | --- | --- | --- | --- | --- | --- |
|  | 1 | 2 | 3 | 4 | 5 | **∑** | 6 | 7 | 8 | 9 | 10 | 11 | 12 | **∑** | **Total** |
| Adams et al. [42] | 1 | 1 | 1 | 1 | 0 | **4** | 3 | 1 | 2 | 1 | 0 | 1 | 1 | **9** | **13** |
| Adams et al. [46] | 1 | 1 | 1 | 1 | 1 | **5** | 2 | 1 | 2 | 1 | 1 | 1 | 1 | **9** | **14** |
| Alizadeh et al. [68] | 1 | 1 | 1 | 1 | 1 | **5** | 1 | 0 | 2 | 0 | 1 | 0 | 1 | **5** | **10** |
| Banerjee et al. [69] | 1 | 1 | 1 | 1 | 1 | **5** | 2 | 0 | 2 | 1 | 0 | 1 | 1 | **7** | **12** |
| Bell et al. [70] | 1 | 1 | 1 | 1 | 0 | **4** | 3 | 1 | 2 | 1 | 1 | 0 | 1 | **9** | **13** |
| Bhatia&Kayser [53] | 1 | 1 | 1 | 1 | 1 | **5** | 2 | 1 | 2 | 1 | 0 | 1 | 1 | **8** | **13** |
| Blackwell et al. [55] | 1 | 1 | 1 | 1 | 1 | **5** | 3 | 1 | 2 | 1 | 0 | 1 | 1 | **9** | **14** |
| Devin et al. [51] | 1 | 1 | 1 | 1 | 0 | **4** | 3 | 0 | 2 | 1 | 1 | 1 | 1 | **9** | **13** |
| Devin et al. [71] | 1 | 1 | 1 | 1 | 0 | **4** | 3 | 0 | 2 | 1 | 0 | 1 | 1 | **8** | **12** |
| Djurhuus et al. [62] | 1 | 1 | 1 | 1 | 0 | **4** | 2 | 1 | 2 | 1 | 0 | 0 | 1 | **7** | **11** |
| Dolan et al. [72] | 1 | 0 | 0 | 1 | 0 | **2** | 3 | 0 | 2 | 1 | 0 | 1 | 1 | **8** | **10** |
| Dunne et al. [73] | 1 | 1 | 1 | 1 | 1 | **5** | 2 | 0 | 2 | 1 | 0 | 1 | 0 | **6** | **11** |
| Hooshmand Moghadam et al. [49] | 1 | 1 | 0 | 0 | 0 | **2** | 1 | 0 | 2 | 1 | 1 | 1 | 1 | **7** | **9** |
| Hwang et al. [74] | 1 | 1 | 1 | 1 | 1 | **5** | 2 | 0 | 2 | 1 | 0 | 1 | 0 | **6** | **11** |
| Kang et al. [75] | 1 | 1 | 1 | 1 | 0 | **4** | 3 | 1 | 2 | 1 | 0 | 0 | 1 | **8** | **12** |
| Karenovics et al. [54] | 1 | 1 | 1 | 1 | 1 | **5** | 2 | 1 | 2 | 1 | 0 | 1 | 1 | **8** | **13** |
| Lee et al. [41] | 1 | 1 | 1 | 1 | 0 | **4** | 2 | 0 | 2 | 1 | 0 | 1 | 1 | **7** | **11** |
| Lee et al. [76] | 1 | 1 | 1 | 1 | 0 | **4** | 2 | 0 | 2 | 1 | 0 | 1 | 1 | **7** | **11** |
| Licker et al. [40] | 1 | 1 | 1 | 1 | 1 | **5** | 1 | 1 | 2 | 1 | 0 | 1 | 1 | **7** | **12** |
| Minnella et al. [50] | 1 | 1 | 1 | 1 | 1 | **5** | 3 | 0 | 2 | 1 | 1 | 0 | 1 | **8** | **13** |
| Northey et al. [77] | 1 | 1 | 1 | 1 | 0 | **4** | 2 | 0 | 2 | 1 | 1 | 1 | 1 | **8** | **12** |
| Ochi et al. [78] | 1 | 1 | 1 | 1 | 1 | **5** | 2 | 0 | 2 | 1 | 1 | 1 | 0 | **7** | **12** |
| Piraux et al. [79] | 1 | 1 | 0 | 1 | 0 | **3** | 3 | 1 | 2 | 1 | 0 | 1 | 1 | **9** | **12** |
| Piraux et al. [80] | 1 | 1 | 0 | 0 | 0 | **2** | 3 | 1 | 2 | 1 | 0 | 1 | 1 | **9** | **11** |
| Reljic et al. [45] | 1 | 1 | 0 | 1 | 0 | **3** | 3 | 0 | 2 | 1 | 1 | 1 | 1 | **9** | **12** |
| Samhan et al. [81] | 1 | 1 | 1 | 1 | 0 | **4** | 2 | 0 | 2 | 1 | 0 | 1 | 1 | **7** | **11** |
| Schmitt et al. [82] | 1 | 1 | 1 | 1 | 0 | **4** | 2 | 0 | 2 | 1 | 1 | 1 | 1 | **8** | **12** |
| Toohey et al. [83] | 1 | 0 | 1 | 1 | 0 | **3** | 3 | 0 | 2 | 1 | 0 | 1 | 1 | **8** | **11** |
| Toohey et al. [84] | 1 | 1 | 1 | 0 | 0 | **3** | 1 | 0 | 2 | 1 | 0 | 1 | 1 | **6** | **9** |
| Toohey et al. [85] | 1 | 1 | 1 | 1 | 0 | **4** | 3 | 0 | 2 | 1 | 0 | 1 | 1 | **8** | **12** |
| Wood et al. [86] | 1 | 0 | 1 | 1 | 1 | **4** | 0 | 0 | 2 | 1 | 0 | 1 | 1 | **5** | **9** |
